# Molecular Approach for the Diagnosis of Blood and Skin Canine Filarioids

**DOI:** 10.3390/microorganisms8111671

**Published:** 2020-10-28

**Authors:** Younes Laidoudi, Samia Bedjaoui, Hacène Medkour, Maria Stefania Latrofa, Abdeslam Mekroud, Idir Bitam, Bernard Davoust, Domenico Otranto, Oleg Mediannikov

**Affiliations:** 1 IRD, AP-HM, Aix Marseille Univ, 19-21 Boulevard Jean Moulin, 13005 Marseille, France; younes.laidoudi@yahoo.com (Y.L.); hacenevet1990@yahoo.fr (H.M.); bernard.davoust@gmail.com (B.D.); 2 Microbes, Evolution, Phylogeny and Infection (MEPHI), IHU Méditerranée Infection, 19-21 Boulevard Jean Moulin, 13005 Marseille, France; 3 PADESCA Laboratory, Veterinary Science Institute, University Constantine 1, El Khroub 25100, Algeria; mekroudabdeslam@gmail.com; 4 Laboratory of Food Hygiene and Quality Insurance System (HASAQ), Higher National Veterinary School, Issad Abbes, Oued Smar, Algiers 16000, Algeria; sbedjaoui@hotmail.com; 5 Department of Veterinary Medicine, University of Bari, 70010 Valenzano, Italy; maria.latrofa@uniba.it (M.S.L.); domenico.otranto@uniba.it (D.O.); 6 IRD, AP-HM, SSA, VITROME, Aix-Marseille Univ, 13385 Marseille, France; idirbitam@gmail.com; 7 Superior School of Food Sciences and Food Industries, Algiers 16004, Algeria; 8 Department of Pathobiology, Faculty of Veterinary Science, Bu-Ali Sina University, Felestin Sq, 6517658978 Hamedan, Iran

**Keywords:** Canine filarioses, *Dirofilaria immitis*, *Dirofilaria repens*, *Cercopithifilariabainae*, *Cercopithifilaria grassii*, *Cercopithifilaria* sp. II, *Onchocerca lupi*, skin, ticks, multiplex qPCR

## Abstract

The zoonotic *Onchocerca lupi* and tick-transmitted filarioids of the genus *Cercopithifilaria* remain less well known due to the difficulties in accessing to skin samples as target tissues. Here, we proposed a molecular approach reliying on multiplex qPCR assays that allow the rapid identification of filarioids from canine blood, skin, and tick samples. This includes two newly developed duplex qPCR tests, the first one targeting filarial and *C. grassii* DNA (CanFil-*C. grassii*). and the second qPCR assay designed for the detection of *Cercopithifilaria bainae* and *Cercopithifilaria* sp. II DNAs (*C. bainae*-C.spII). The third one is a triplex TaqMan *cox 1* assay targeting DNA of blood microfilariae (e.g., *Dirofilaria immitis, Dirofilaria repens* and *Acanthocheilonema reconditum*). The novel duplex qPCRs developed were validated in silico and by screening of known DNA collection. The qPCR assays were also used for screening the blood and tick samples of 72 dogs from Algeria. This allowed the identification of canine filariasis infection with 100% of specificity and 89.47% and 100% of sensitivity from naturally infected blood and tick samples, respectively. The prevalences of 26.39% for *D. immitis* and 5.56% for both *D. repens* and *A. reconditum* were reported in blood and tick samples. *Cercopithifilaria* DNAs were detected only in tick samples, with a prevalence of 4.17% and 5.56% for *C. bainae* and *Cercopithifilaria* sp. II, respectively. Co-infections were diagnosed in 6.94% and 13.89% of blood and tick samples, respectively. Whereas all samples were negative for *C. grassii* DNA. The use of engorged ticks instead of blood and skin samples could be an easier option for the surveillance of all canine filarioids herein investigated. The multiplex qPCR assays herein validated were shown to be useful in the detection of filarial co-infections by overcoming sequencing of positive samples.

## 1. Introduction

Canine filarioses are a group of diseases caused by arthropod-borne filarioids (Spirurida: Onchocercidae) belonging to the genera *Dirofilaria*, *Acanthocheilonema, Cercopithifilaria, Brugia*, and *Onchocerca* [1,2,3]. In addition to their veterinary importance, many of them are zoonotic. The adult filarioids live in different districts from the hearth (*Dirofilaria immitis*) to the ocular cavities (*Onchocerca lupi*) and, many of them, in the subcutaneous tissues (i.e., *Cercopithifilaria grassii, Cercopithifilaria* sp. I *and Cercopithifilaria* sp. II, *Dirofilaria repens* and *Acanthocheilonema reconditum*). Once mature, the viviparous nematodes produce blood or cutaneous microfilariae (mfs), which are available to an arthropod vectors for their cycle to complete to the infective third stage larvae [1]. The availability of the microfilariae in different animal tissues and anatomical regions is related to their detection [4] and, therefore, to their diagnosis. For example, microfilariae in the subcutaneous tissues such as *Cercopithifilaria* spp. and the zoonotic *O. lupi* are less diagnosed or completely non-diagnosed in comparison to those circulating in blood such as *D. immitis, D. repens, A. reconditum* and *Acanthocheilonema dracunculoides,* where they are routinely diagnosed by several assays such as morphological identification, molecular and serological tests [5]. Recently, great importance has been given to cutaneous filariases caused by *O. lupi* and *Cercopithifilaria* spp. [6,7,8,9]. These latter are transmitted by hard ticks belonging to Ixodidae family [10], whilst for the first one pathogen the vector is still unknown. *Onchocerca lupi* was firstly detected from a Caucasian wolf (Canis lupus) in Georgia [11] and subsequently diagnosed in domestic animals (i.e., dogs and cats) from European countries (i.e., Hungary, Greece, Germany and Portugal) and USA [12,13,14,15,16,17,18,19]. At present, the diagnosis of *Cercopithifilaria* spp. and *O. lupi* is based mainly on microscopic examination of dog skin snip sediments and the identification of adults embedded in cutaneous/ocular nodules [3]. However, this method is quite invasive since it requires a skin biopsy, therefore representing a major limitation to this diagnosis technique in the clinical routine [20]. Molecular techniques have recently been standardized for the detection of *O. lupi* DNA [3].

After the first description of *Cercopithifilaria grassii* in 1907 by Noè in dogs in Italy, this filariasis remained mysterious until 1982, when larvae of a *Cercopithifilaria* spp. were observed in ixodid ticks in Switzerland [21], then in the brown dog ticks (*Rhipicephalus sanguineus*) in northern Italy [22]. In 1984, Almeida and Vicente managed to identify another cutaneous canine filarial species, *Cercopithifilaria bainae*. Later, Otranto et al. (2011), reported the same species from a Sicilian dog and gave it the name *Cercopithifilaria* sp. I. Subsequently, the same author provided the full description of the species and the name *Cercopithifilaria bainae* was formally retained [10]. A third species of *Cercopithifilaria* sp. mfs have been identified as *Cercopithifilaria* sp. II as a formal description was not carried out since adult specimens have never been detected [10,23]. In 2012, Otranto et al. have morphologically and molecularly characterized *C. grassii* and *Cercopithifilaria* sp. II mfs from samples derived from European dogs. This extensive study has shown that dogs can be parasitized by three dermal species namely, *C. grassii*, *C. bainae,* and *Cercopithifilaria* sp. II [7,24]. In 2014, Solinas et al. conducted a study whose objective was to determine the genetic constitution of *C. bainae* and *Cercopithifilaria* sp. II. [25].

To improve the molecular diagnosis of canine filariasis and to better understand the interactions of the filarioids among them, we propose in this study a novel multiplex qPCR approach. It consists primarily of two duplex and one triplex TaqMan *cox*-1-based qPCR assays for the simultaneous detection and differentiation of *D. immitis, D. repens, A. reconditum, C. grassii, C. bainae,* and *Cercopithifilaria* sp. II DNA. The approach was completed by PCR/sequencing assay to detect the other canine filarioids having blood and skin mfs. Secondly, the approach was standardized on ticks infesting dogs as a suitable sample to molecularly explore all etiological agents of canine filariasis.

## 2. Materiel and Methods

### 2.1. Design Protocol and Specificity-Based Principles of the Duplex Real Time qPCRs

The mitochondrial gene encoding for the cytochrome *c* oxidase subunit 1 (*cox* 1 gene) was targeted for its presence in several copies by cell and described as a “barcode gene” for filarial nematodes [26]. PCR design was performed according to the criteria for primers and probes protocol [27]. Briefly, primers and probes of two duplex qPCR assays (Table 1) targeting filarial nematodes and *C. grassii* DNA (i.e., CanFil-*C.grassii*) and those of *C. bainae* and *Cercopithifilaria* sp. II (i.e., *C. bainae*-*C.* sp. II), were designed by alignment of sequences from representative members of Onchocercidae family available from GenBank database, using primer3 software v. 0.4.0 (http://primer3.ut.ee). Subsequently, all possible combinations of forward-reverse and probe-reverse of each qPCR system were checked within the DNA databases of metazoans (taxid:33208), vertebrates (taxid:7742), bacteria (taxid:2), Canidae (taxid:9608), Felidae (taxid:9682), and humans (taxid:9605) using primer-BLAST [28]. Primers and hydrolysis probes were synthetized by Eurogentec (Liège, Belgium) and Applied Biosystems^TM^ (Foster City, CA, USA), respectively.

### 2.2. Run Protocols

Both duplex qPCR reactions were carried out in a total volume of 20 µL. The reaction mixture contained 10 µL of Master Mix Roche (Eurogentec), 2 µL of ultra-purified water DNAse-RNAse free, 0.5 µL of each primer (20 µM of concentration), and 0.5 µL for both UDG and each probe (5 µM of concentration). Finally, 5 µL of DNA template was added to the mixture. The TaqMan cycling protocol included two hold steps at 50 °C for 2 min followed by 15 min at 95 °C, and 39 cycles of two steps each (95 °C for 30 s and 60 °C for 30 s). These reactions were performed in a thermal cycler CFX96 Touch detection system (Bio-Rad, Marnes-la-Coquette, France).

### 2.3. Assays Validation

#### 2.3.1. Specificity Validation

Duplex-qPCR assays were challenged against two DNA collection: (i) A total of 46 genomic DNA, consisting of 34 samples from adult or larval filarioids from 14 species (i.e., *Cercopithifilaria* sp. II, *C. grassii, C. bainae, Onchocerca lupi, Dirofilaria immitis, D. repens, Acanthocheilonema reconditum, Setaria digitata, Mansonella* sp., *M. perstans, Wuchereria bancrofiti, Loa loa, Brugia*sp., *B. malayi*), 2 DNA samples from the thelazioid eye worm (*T. callipaeda*), and 10 filarial free samples (5 skin samples and 5 ticks), and (ii) 57 DNA samples (Supplementary Table S1) that consist of non-filarial nematodes, arthropods, laboratory-maintained colonies, as well as human, monkey, donkey, horse, cattle, mouse, and dog DNAs.

#### 2.3.2. Limit of Detection and Efficiency Assessment

The analytical sensitivity of the newly developed qPCRs was assessed using a serial 10-fold dilutions of both single-species and spiked DNAs. The DNA of *O. lupi* and *C. grassii* as well as the spiked DNA from both species were used for the duplex qPCR CanFil-*C. grassii* while the DNA from *C. bainae* and *Cercopithifilaria* sp. II and the spiked DNA samples were used for the duplex qPCR *C. bainae-C.* sp. II. The sensitivity of each assay was assessed by generating the standard curves and by analyses of the derived parameters (i.e., efficiency, slope, Y-intercept, and correlation coefficient) within CFX Manager Software Version 3 [29].

#### 2.3.3. Microfilariae Quantification Protocol

The quantification protocol has been performed for the duplex qPCR filaria-*C. grassii* to evaluate the detection limit in term of mfs concentration from biological samples. A serial 10-fold dilutions of *D. immitis* DNA extracted from 200 µL of infected canine blood [5] containing 470 mfs per mL (i.e., 94 mf/200 µL of eluted DNA and 2.35 mfs/5 µL of qPCR reaction) were tested. In addition to the standard curves, the relative fluorescence units (RFUs) from the dye (VIC, Table 1) were used to evaluate the qPCR efficiency in detecting the related mfs DNA as previously described [5]. The cut-of value was determined with a tolerance coefficient of 5% according to the formula described [29].

### 2.4. Set Up of a Molecular Approach for the Diagnosis of Blood and Skin Filarioids 

#### 2.4.1. Samples Collection 

During an expedition to the Northern Algeria canine samples (blood and ectoparasites) have been collected [30]. The study area was known to be endemic for ticks and tick-borne pathogens such as [30] *Rickettsia massiliae, Rickettsia conorii,* and *Ehrlichia canis* [31]. A total of 567 ticks were collected from 72 (32%) dogs out of 227 animals sampled [30]. Ticks from each dog were kept in tubes containing 70° of ethanol and were conducted to our laboratory for further analysis. One engorged tick and blood samples of each infested dog (*n* = 72) were subjected to molecular analysis.

Genomic DNA was extracted individually from all tick body and blood samples. In order to minimize PCR inhibitors from tick samples, we followed the extraction protocol described by Halos et al., 2004 [32]. Briefly, a bead-based physical disruption of the tick body within the Tissue-Lyser apparatus (Qiagen, Hilden, Germany), and 24 h of enzymatic digestion at 56 °C using buffer G2 supplemented with 25% of proteinase K were performed prior DNA extraction. Meanwhile, blood samples were subjected only to the enzymatic digestion prior to DNA extraction. The extraction was performed using the EZ1 DNA tissue kit (Qiagen, Courtaboeuf, France), in line with the manufacturer’s instructions. DNA was eluted in a final volume of 200 µL and stored at −20 °C until analysis.

#### 2.4.2. Ethics Approval and Consent to Participate

Dogs were examined by veterinarians with the assistance and acceptance of their owners. Ethical aspects related to dog sampling were treated in accordance with Algerian legislation guidelines. Risk assessment was submitted to and approved by the ethics committee and decision board of the veterinary practitioners from the wilayas of the North of Algeria. These institutions are affiliated with the Algerian Ministry of Agriculture and Rural Development (Directions des Services Vétérinaires). Protocol of the study was also approved by the scientific college (Procès-Verbal du CSI N°6, 2018) at the Veterinary Science Institute, University Constantine 1, Algeria. To facilitate field work, collaborations were established with veterinary doctors and their assistants working in these establishments.

#### 2.4.3. Diagnostic Approach Standardization on Biological Samples

To gain further insight into the diagnosis value of filarial infection from canine samples, we assessed a multiplex qPCRs approach based on two duplexes qPCRs (CanFil-*C. grassii* and Cerco spI-spII) herein developed and another multiplex qPCR system (Triplex TaqMan *cox* 1) [5]. The approach was proposed to explore the presence of filarial DNA followed by species-level identification of *C. grassii, C. bainae, Cercopithifilaria* sp. II, *D. immitis, D. repens* and *A. reconditum*. The amplification and sequence typing approach using filaria generic PCR primers and probes [Pan-fil *cox* 1 PCR] [5] targeting the partial (509 bp) *cox* 1 gene of filarial nematodes have been used. Sequencing analysis was performed to achieve the identification at the species level. Briefly, PCR positive products were resolved in 0.5x GelRed stained (Biotium, Fremont, CA, USA) agarose gels (2%), then purified using NucleoFast^®^ (Macherey Nagel, Düren, Germany) 96 PCR DNA purification plate prior to run on the BigDye™ Terminator v3.1 Cycle Sequencing Kit (Applied Biosystems, Foster City, CA, USA). The BigDye products were purified on the Sephadex G-50 Superfine gel filtration resin (Cytiva, Formerly GE Healthcare Life Sciences, Sweden) prior the sequencing on the ABI Prism 3130XL (Applied Biosystems, Foster City, CA, USA). Finally, *cox* 1 nucleotide sequences were edited using ChromasPro 2.0.0 (Technilysium Pty Ltd., Brisbane, Australia), aligned against the closely related species using MAFFT [33]. Best fit model and maximum likelihood phylogeny were performed on MEGA 6 [34]. Phylogram was edited using iTOL v4 software [35].

Standardization of the assays was performed on two different panels of canine samples (i.e., blood and tick samples). However, because the gold standard tests (dog necropsy, blood concentration test and tick dissection) were absent, filarial true-positive dogs were considered if at least one DNA sequence was obtained from at least one sample (i.e., tick or blood) of each dog. However, samples that had yielded unreadable DNA sequences (overlapping peaks in the electropherograms) from both tick and blood samples of the same dog were removed from the analysis. Finally, prevalence, correct classification, misclassification, sensitivity, specificity, false positive rate, false negative rate, positive and negative predictive value, and Youden index were calculated for each approach [36,37,38].

## 3. Results

The in-silico and in-vitro validations revealed that, the newly designed duplex-qPCRs were specific for the target species. The duplex COI-based qPCR for *C. grassii* and canine filarioids amplified all filarial species (*n* = 14) and the thelazioid nematode (*T. callipaeda*) assessed from different biological sources discriminating *C. grassii* DNA (Table 2). The duplex COI-based qPCR for *Cercopithifilaria* spp. was able to detect and discriminate between *C. bainae* and *Cercopithifilaria* sp. II. No DNA amplification was obtained from both free-filarial tick and skin samples as well as from the panel of negative controls summarized in Supplementary Table S1.

### Analytical Sensitivity and Assay Performance Characteristics 

The analytical sensitivity of each duplex qPCR was confirmed by qPCR efficiencies values ranging from 99.3% to 104.9%, with slope from −3.34 to −3.21, Y-intercept values from 40.541 to 45.792 with an R^2^ ≥ 0.99 for all qPCR reactions (Table 3, Supplementary Figure S1 and S2).

Results of the detection limit of the duplex qPCR targeting canine filarioids and *C. grassii* are detailed in Table 4. The assay was able to detect up to 4.7 × 10^−2^ mfs/mL (i.e., corresponding to 2.35 × 10^−4^ mfs/5 μL) with an efficiency of 99.8% and a slope of −3.327 and with a perfect adjustment (R^2^ = 0.999) (Supplementary Figure S3).

**Figure 1:**
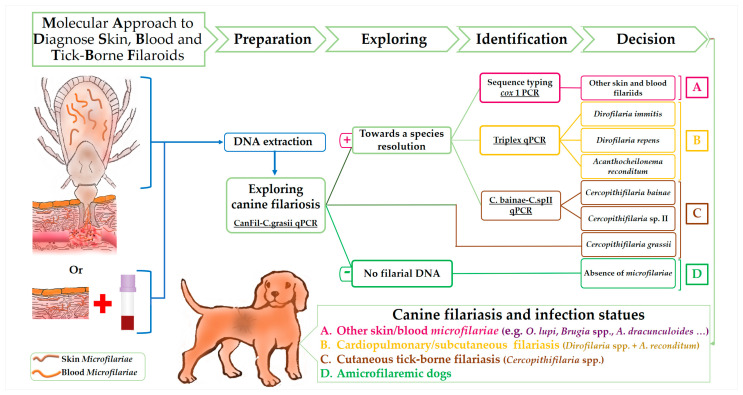
Flow Diagram showing the specificity-based principles of the proposed molecular approach in detecting canine filaroids.

The following figure (Figure 1) shows the suitable samples and the principles of multiplex qPCRs design based on specificity in the current approach to diagnose canine filariasis. PCR systems are classed according to their specificity from exploring to species-specific resolution.

The detailed results of molecular identification of filarial DNA from blood and tick samples are shown in Table 5. Overall, the multiplex qPCR approach allowed the identification of 22 (30.55%) filarial-positive samples. In particular, 14 (19.44%), two (2.78%) and one (1.39%) blood samples were positive for *D. immitis, D. repens* and *A. reconditum,* respectively, and five (6.94%) were coinfected, two (2.78%) with *D. immitis* and *D. repens* and three (4.17%) with *D. immitis* and *A. reconditum*. All blood samples were negative for *Cercopithifilaria* spp. Accordingly, all filarial species identified in the blood of dogs were also detected in their ticks. Whilst, *Cercopithifilaria* spp. DNA was found in six ticks, four (5.56%) *D. immitis* positive samples and one (1.39%) *D. immitis-D. repens* coinfected sample were also positive for *C. bainae*. *Cercopithifilaria* sp. II was detected in 3 (4.14%) ticks, one among them was also positive for *D. immitis*.

**Figure 2:**
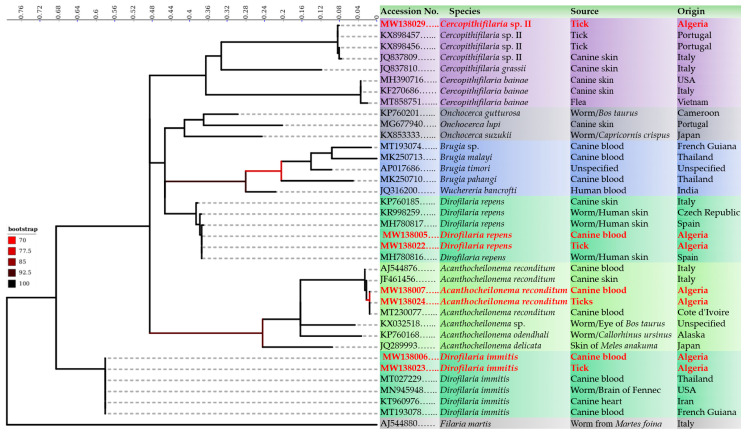
Phylogenetic tree showing the clusterization of filarial genotypes identified in the present study with the other filarioids. The tree was inferred using the Maximum Likelihood method based on 1000 bootstraps and the Tamura-Nei model. The analysis involved 36 partials (453 bp) *cox* 1 sequences of filarioids. Outgroup taxons *Filaria martis* (AJ544880) is drawn at root. A discrete Gamma distribution was used to model evolutionary rate differences among sites (5 categories (+G, parameter = 0.5779)). The rate variation model allowed for some sites to be evolutionarily invariable ([+I], 49.5935% sites). Log likelihood was-2738.5083. The axis showed the global distance observed throughout the trees. The identity of each taxa is color-coded according to the genus. Branches are color-coded according to the bootstraps percent.

Accordingly, all filaria-positive samples by the qPCR were also amplified by filaria generic PCR [Pan-fil *cox* 1 PCR]. High quality DNA sequences were obtained from 17 (23.61%) blood samples, including 14 (19.44%) *D. immitis,* two (2.78%) *D. repens* and one (1.39%) *A. reconditum* and 14 (19.44%) tick samples, including nine (12.5%) *D. immitis,* two (2.78%) *D. repens*, one (1.39%) *A. reconditum* and two (2.78%) *Cercopithifilaria* sp. II. Whilst five (6.94%) and 10 (13.89%) samples have yielded unreadable DNA sequences (overlapping peaks in the electropherograms) from blood and ticks respectively (Table 5). Phylogenetic analysis confirmed the molecular identification of each filarial detected from blood and/or tick samples by clustering the representative sequences within the clades of the same reference species (Figure 2). DNA sequences generated during the present study were deposited in GenBank under the accession number from MW138005 to MW138035.

Despite the successful species resolution of the multiplex approach from tick and blood samples of all filarial positive dogs (*n* = 24), specificity and sensitivity analysis involved only 19 among them (Supplementary Table S2). These latter were considered as filarial true positives dogs, from which at least one DNA sequence was obtained from their blood and/or tick samples (Supplementary Table S2). A total of 48 dogs were negative for filarial DNA from both blood and tick samples. Five dogs were excluded from the analysis because they were coinfected and have yielded unreadable DNA sequences (overlapping peaks in the electropherograms) from their blood and tick samples.

Compared to the gold standard (Table 6), the multiplex qPCRs approach combining the identification of *D. immitis, D. repens, A. reconditum, C. grassii, C. bainae*, and *Cercopithifilaria* sp. II allowed the diagnosis of canine filarioids with 100% of specificity in 89.47% and 100% of cases from their blood and ticks respectively (Youden index of 0.86 and 1, respectively). While the sequence typing approach allowed the diagnosis of canine filariasis with 100% of specificity in 89.47% and 73.68% of cases from their blood and ticks respectively (Youden index of 0.86 and 0.74, respectively).

## 4. Discussion

In this study we assessed a molecular approach relaying on multiplex qPCR assays that allow the rapid identification of filarioids from canine blood, skin, and tick samples. Canine filarial agents such as the zoonotic *O. lupi* and tick-transmitted filarioids of the genus *Cercopithifilaria* speread in many areas of Europe, Mediterranean Basin and several part of the world [39,40]. Furthermore, their life cycle, vectors as well as the parasites themselves are less or completely unknown for some of them [3,7]. Depending on monitoring progress and vector surveillance, their detection contributes in avoiding the introduction and/or spread of these vector-borne helminths causing diseases [41]. Therefore, considering the zoonotic role for some of these parasites with an increasing of the public health implications, the diagnosis and/or the monitoring assays must provide species-level identification to properly assist in decisions for medical and preventive treatments. The identification of filarial agent provides a better understanding on the distribution and prevalence of the disease. In addition to the poorly developed veterinary diagnostic services [41], diagnosing skin filarial agents remains laborious and difficult because of the limited access to skin samples and parasite material. As a consequence, the approaches for managing these health threatening parasites might be incomplete and need more development.

Molecular detection of the majority of canine filariasis of the genus *Cercopithifilaria* relies heavily on sequence typing method. This method is based on the use of filaria generic primers for DNA amplification and sequencing analysis [8,42], since filaria generic primers can theoretically amplify any filarial DNA [5]. However, as we demonstrated here and elsewhere [5,43], the sequence typing method may not allow species identification when the samples are coinfected with two or more species. Our findings showed that, the new duplex-qPCRs (CanFil-*C. grassii* and C. bainae-CspII) were specific to the target species without failure. A high analytical sensitivity was provided by each duplex qPCR in detecting single-species and/or pooled DNA with an efficiency ranged from 99.3% to 104.9% and a coefficient of determination (R^2^) greater than 0.99. Furthermore, the duplex CanFil-*C. grassii* also explore the presence of filarial DNA, which assist in decision for further investigations and allowed rapidly information about the presence/absence of filarial DNA, an important step when the diagnosis approach relies on several species-specific assays. Although when used together, the novel duplex qPCRs and the triplex TaqMan *cox* 1 assays allowed the identification of canine filariasis caused by *C. bainae*, *Cercopithifilaria* sp. II, *D. immitis, D. repens* and *A. reconditum* with 100% of specificity and 89.47% and 100% of sensitivity from naturally infected blood and tick samples, respectively. These features were higher than those of the sequence typing approach, which consolidates the usefulness of multiplex qPCR in the detection of filarial co-infections and reinforces the previous studies [5,44].

Another limitation of either molecular or parasitological diagnosis of canine filariasis is the choice of samples, since both methods are often targeting mfs specimens. However, these larvae have different locations in the host. Indeed some of them such as *D. immitis*, *D. repens* and *A. reconditum* are located in the bloodstream with a different density, while *Cercopithifilaria* spp. and other skin mfs are distributed unevenly in superficial dermal tissues [4]. The only two reports of the blood *C. bainae* DNA remain inconclusive and could just be an accidental detection of micro-fragments from dead microfilaria [43,45]. These features indicating that the exhaustive diagnosis of canine filariasis should rely on both blood and skin samples.

Data presented here demonstrated that, tick samples are more suitable for exploring both blood and skin microfilaria when the assay is able to discriminate at the species-level the coinfections. In addition to their role as vector for *Cercopithifilaria* spp. [25], tick are co-evolved with these filarioids and shown the same predilection sites on their hosts (dogs) [4], which indicates the close contact of *Cercopithifilaria* microfilaria and ticks within infected dogs. The recent study of Lineberry et al., (2020), reported the DNA of *C. bainae* in ticks infesting positive dogs [42]. Furthermore, several studies reported the presence of filarial DNA from ticks infesting animals [46,47], indicating that ticks could be considered as equivalent to blood sample in detecting filarioids. The use of the hematophagous arthropod as an alternative blood sampling method was demonstrated for Triatomine bugs. This sampling method was advantages in obtaining blood samples without anaesthesia from animals where veins are inaccessible [48]. Notwithstanding the absence of skin from sample panels herein tested, which may represent a limitation of the multiplex qPCR approach, the use of ticks infesting dogs provides an alternative to the complicated sampling methods requiring both blood and skin samples from the same dogs. Thereby, exploring filarial DNA from engorged ticks offers the possibility to detect both skin and blood mfs and reduces the sampling and analyzing steps.

In this study, we observed 29% and 33% prevalence of filarial infections from blood and tick samples from Algerian dogs, respectively, which are almost identical to those previously observed in dog blood samples from India (26.5%) [49] and from Italy (23%) [50,51]. In addition to *D. immitis* already described, for the first time, we report the presence of *D. repens, A. reconditum, C. bainae* and *Cercopithifilaria* sp. II in Algeria. Here the prevalence observed for *D. immitis* was 23.61%, which is close to that reported from the same study area (Northern Algeria) by Ben-Mahdi and Madani (2009), where 24.46% of dogs were *D. immitis*-antigens positive [52], but it was higher than that reported by Tahir et al. in 2015, who reported a prevalence of 1.4% for *D. immitis* in dog blood samples by molecular tests [53]. A very high prevalence of canine microfilaraemia of 42.68% was observed in Cherthala in the state of Kerala, a southern area of India [2]. In Northern Virginia, 0.74% *Amblyomma americanum* ticks carried filarial nematode DNA [54]. In Southern Connecticut, infection rate of *Acanthocheilonema* filarial nematode in *Ixodes* ticks is relatively high with 22% and 30% in nymph and adult ixodid ticks, respectively [46]. The overall prevalence of *Cercopithifilaria* sp. in the sampled animal populations was 13.9% and 10.5% by microscopy of skin sediments and by PCR on skin samples, respectively. The higher prevalence rate of infested animals was recorded in Spain either by microscopical examination of skin sediments (21.6%) or by molecular detection on skin samples (45.5%) whereas the lower positivity rate was in Greece (4.3%). In Italy, according to the sites and to the diagnostic tests employed, the prevalence of *Cercopithifilaria* spp. infestation in dogs varied from 5.3% up to 19.5% [10]. Differences in reported prevalence levels among studies may due to diagnosis tool performances, the different tissues sampled, the number of animals tested, but also due to the geographical distribution of tick vectors transmitting pathogens.

## 5. Conclusion

The diagnosis approach combining species-specific multiplex qPCR assays allowed the identification of *D. immitis, D. repens, A. reconditum, C. grassii, C. bainae,* and *Cercopithifilaria* sp. II despite the presence of coinfection. The use of ticks infesting dogs instead of blood and skin samples could be an easier way that contribute to disease progress monitoring and to the surveillance of canine filariasis. This would be particularly relevant, since most of them are pathogenic for dogs and constitutes an emergent zoonosis. Although the new qPCR assays standardized for specific detection of *Cercopithifilaria* species may ultimately assist in the quest to identify the elusive adult *Cercopithifilaria* sp. II. We demonstrated the pressure caused by canine vector-borne filariasis and how intense the challenge was for dogs in Algeria. We draw general attention to public health risks, since dogs are sentinels for potential zoonosis. There is an urgent need for the implementation of preventive strategies against canine vector filarioids. Finally, we encourage researchers to follow the molecular procedure summarized in Figure 1 to explore, diagnose, and monitor canine filariasis from ticks infesting dogs unless combining blood and skin samples.

## Figures and Tables

**Table 1:** Primers and Probes Designed for Duplex qPCR Assays accordingly to Species Examined.

Assay Name	Sequences Names	Sequences	Specificity
**CanFil-*C. grassii***	C. Fil.354f	GATCGTAATTTTARTACYTCTTTTTATGA	Filaria/*C. grassii*
	*Can-fil*.411p	6VIC-TATCAGCATTTGTTTTGGTTTTT-TAMRA	
	*C. grassii*.433p	6FAM-GGAAGGGTGGTAATCCTCTTCTTT-TAMRA	
	C. Fil.564r	CAGCAATCCAAATAGAAGCAAA	
***C. bainae*-C.spII**	T-Fil-62f	TTGTCTTTTTGGTTTACTTTTGTGG	*C. bainae/Cercopithifilaria* sp. II
	*C. bai*.121p	6FAM-AGGGGGTGCTGGTAGCAGG-TAMRA	
	*C.* sp. II.116p	6VIC-GTTGGTAGAGGCCCTGGGAGT-TAMRA	
	T-Fil-337r	GAAGTCAAATAAGAAGTRCAAACAAACA	

**Table 2:** In-vitro validation of the newly customized duplex qPCRs trough the DNA panel of filarioids and negative hosts (dogs and ticks).

Panels of Tested DNA from Filarioids			CanFil-*C. grasii*		*C. bainae-C.* sp II	
Species Name	Specimens	Tested Samples (*n*)	Filarial DNA	*C. grassii*	*C. bainae*	*C.* sp. II
***C. bainae***	Adult worms	1	1	Negative	1	Negative
	Larva F1 “Microfilaria”	2	2	Negative	2	Negative
	Infected ticks	5	5	Negative	5	Negative
***Cercopithifilaria* sp. II**	Larva F1 “Microfilaria”	1	1	Negative	Negative	1
***C. bainae + C.*** **sp. II**	Mixed DNA	3	3	Negative	3	3
***C. grassii***	Larva F1 “Microfilaria”	1	1	1	Negative	Negative
***C. grassii + C. bainae***	Mixed DNA	3	3	3	3	Negative
***C. grassii + C.*** **sp. II**	Mixed DNA	2	2	2	Negative	2
***O. lupi***	Larva F1 “Microfilaria”	1	1	Negative	Negative	Negative
	Infected skin	6	6	Negative	Negative	Negative
***D. immitis***	Adult worms	2	2	Negative	Negative	Negative
***D. repens***	Adult worms	1	1	Negative	Negative	Negative
***A. reconditum***	Blood microfilaria	1	1	Negative	Negative	Negative
***T. callipaeda***	Adult worms	2	2	Negative	Negative	Negative
***S. digitata***	Adult worms	2	2	Negative	Negative	Negative
***Mansonella sp.***	Blood microfilaria	1	1	Negative	Negative	Negative
***M. perstens***	Blood microfilaria	1	1	Negative	Negative	Negative
***W. bancrofiti***	Blood microfilaria	2	2	Negative	Negative	Negative
***Loa loa***	Blood microfilaria	1	1	Negative	Negative	Negative
***Brugia* sp.**	Infected mosquitoes	3	3	Negative	Negative	Negative
***B. malayi.***	Infected mosquitoes	3	3	Negative	Negative	Negative
**Filarial free samples**	Ticks	5	Negative	Negative	Negative	Negative
	Dog skin	5	Negative	Negative	Negative	Negative

**Table 3:** Analytical Sensitivities and Performance Characteristics of the Duplex qPCRs in Detecting Single Species and Pooled DNAs.

Assays	DNA Target		Efficiency (%)	Coefficient of Determination (R^2^)	Slope	Y-Intercept
**CanFil-C.grasii**	Single-species DNA	*Onchocerca lupi*	103.2	0.994	−3.247	40.541
		*Cercopithifilaria grassii*	99.3	0.999	−3.38	41.018
	Pooled DNAs	*O. lupi*	103.8	0.993	−3.235	43.74
		*C. grassii*	99.3	0.996	−3.34	45.107
**C. bainae-C.sp.II**	Single-species DNA	*Cercopithifilaria bainae*	100.3	0.996	−3.314	43.902
		*Cercopithifilaria* sp. II.	100.3	0.997	−3.315	45.792
	Pooled DNAs	*C. bainae*	99.5	0.995	−3.334	44.918
		*Cercopithifilaria* sp. II.	104.9	0.994	−3.21	43.694

**Table 4:** Sensitivity and Assay Performance Characteristics of the Duplex COI-based System in Detecting *D. immitis* Microfilariae.

SQ mfs/mL	SQ Per qPCR Reaction from mfs/5µL	Ct Mean	E-RFU	SCRS
**4.7 × 10^+2^**	2.35 × 10^0^	24.26	1554	[E = 96.9%] [S = −3.398] [Y.int = 33.2] [R^2^ = 0.998]
**4.7 × 10^+1^**	2.35 × 10^−1^	27.35	1363	
**4.7 × 10^0^**	2.35 × 10^−2^	31.03	1046	
**4.7 × 10^−1^**	2.35 × 10^−3^	34.02	596	
**4.7 × 10^−2^**	2.35 × 10^−4^	37.92	175	
**4.7 × 10^−3^**	2.35 × 10^−5^	N/A	N/A	
**4.7 × 10^−4^**	2.35 × 10^−6^	N/A	N/A	
**4.7 × 10^−5^**	2.35 × 10^−7^	N/A	N/A	
**4.7 × 10^−6^**	2.35 × 10^−8^	N/A	N/A	
**4.7 × 10^−7^**	2.35 × 10^−9^	N/A	N/A	
**Cut Off Value**		38.0	161	
**Negative Control**		N/A	6.09	

**SQ:** Starting Quantity, **mfs**: microfilaria, **Cq**: cycle quantification value; **N/A**: No amplification, **E-RFU**: End of relative fluorescence unit, **SCRS**: Standard Curve Results Spreadsheet, **E**: Efficiency, **S**: Slope, **Y.int:** Y-intercept.

**Table 5:** Distribution of Positive Samples Detected by Each Molecular Assay (i.e., Multiplex qPCRs and PCR/Sequencing) from Canine Blood and Tick Samples.

Filarial Species	Multiplex qPCR		PCR/Sequencing	
	Blood	Tick	Blood	Tick
**Single-species DNA**				
*D. immitis*	14	9	14	9
*D. repens*	2	2	2	2
*A. reconditum*	1	1	1	1
*C. bainae*	0	0	0	0
*Cercopithifilaria* sp. II	0	2	0	2
Total	17	14	17	14
**Multi-species DNA**				
*D. immitis* and *D. repens*	2	1	2 ur	1 ur
*D. immitis* and *A. reconditum*	3	3	3 ur	3 ur
*D. immitis* and *C. bainae*	0	4	0	4 ur
*D. immitis* and *Cercopithifilaria* sp. II	0	1	0	1 ur
*D. immitis*, *D. repens* and *C. bainae*	0	1	0	1 ur
Total	5	10	5 ur	10 ur

ur: PCR positive samples that yielded unreliable DNA sequences.

**Table 6:** Performance Characteristics of Molecular Assays in Identifying Filarial DNA from Canine Blood and Ticks.

Performances (in % Unless Specified)	Multiplex qPCRs Approach		Sequence Typing Approach	
	Ticks	Blood	Ticks	Blood
True positive (*n* = 19)	19	17	14	17
True negative (*n* = 48)	48	48	48	48
False positive (*n*)	0	0	0	0
False negative (*n*)	0	2	5	2
Total	67	67	67	67
Sensitivity	100	89.47	73.68	89.47
Specificity	100	100	100	100
Predictive positive value (PPV)	100	100	100	100
Predictive negative value (PNV)	100	96	90.57	96
False positive rate	0	0	0	0
False negative rate	0	4	9.43	4
Correct classifcation	28.36	28.36	28.36	28.36
Prevalence	28.36	25.37	20.9	25.37
Youden index	1	0.86	0.74	0.86

## Supplementary Materials

The following are available online at https://www.mdpi.com/2076-2607/8/11/1671/s1. Table S1: Negative control DNA used for specificity determination of designed qPCR systems; Table S2: Molecular identification of filarial DNA from positive samples (tick/blood). Figure S1: Analytical sensitivity of the CanFil-*C. grassii* assay in detecting (a) filarial DNA (i.e., *Onchocerca lupi*), (b) *Cercopithifilaria grassii* and (c) pooled DNA of *O. lupi* and *C. grassii*. Figure S2: Analytical sensitivity of the C. bainae-C.sp.II assay in detecting (a) *Cercopithifilaria* bainae, (b) *Cercopithifilaria* sp. II and (c) pooled DNA of *C. bainae* and *Cercopithifilaria* sp. II. Figure S3: Analytical sensitivity and detection limit of the CanFil-*C. grassii* assay using a serial 10-fold dilution of single-species-DNA of *Dirofilaria immitis microfilariae*.
