# Supplementary figures and images for: Molecular Approach for the Diagnosis of Blood and Skin Canine Filarioids
Source: Microorganisms. 2020 Oct 28;8(11):1671. doi: 10.3390/microorganisms8111671 (PMC7713008; doi:10.3390/microorganisms8111671)

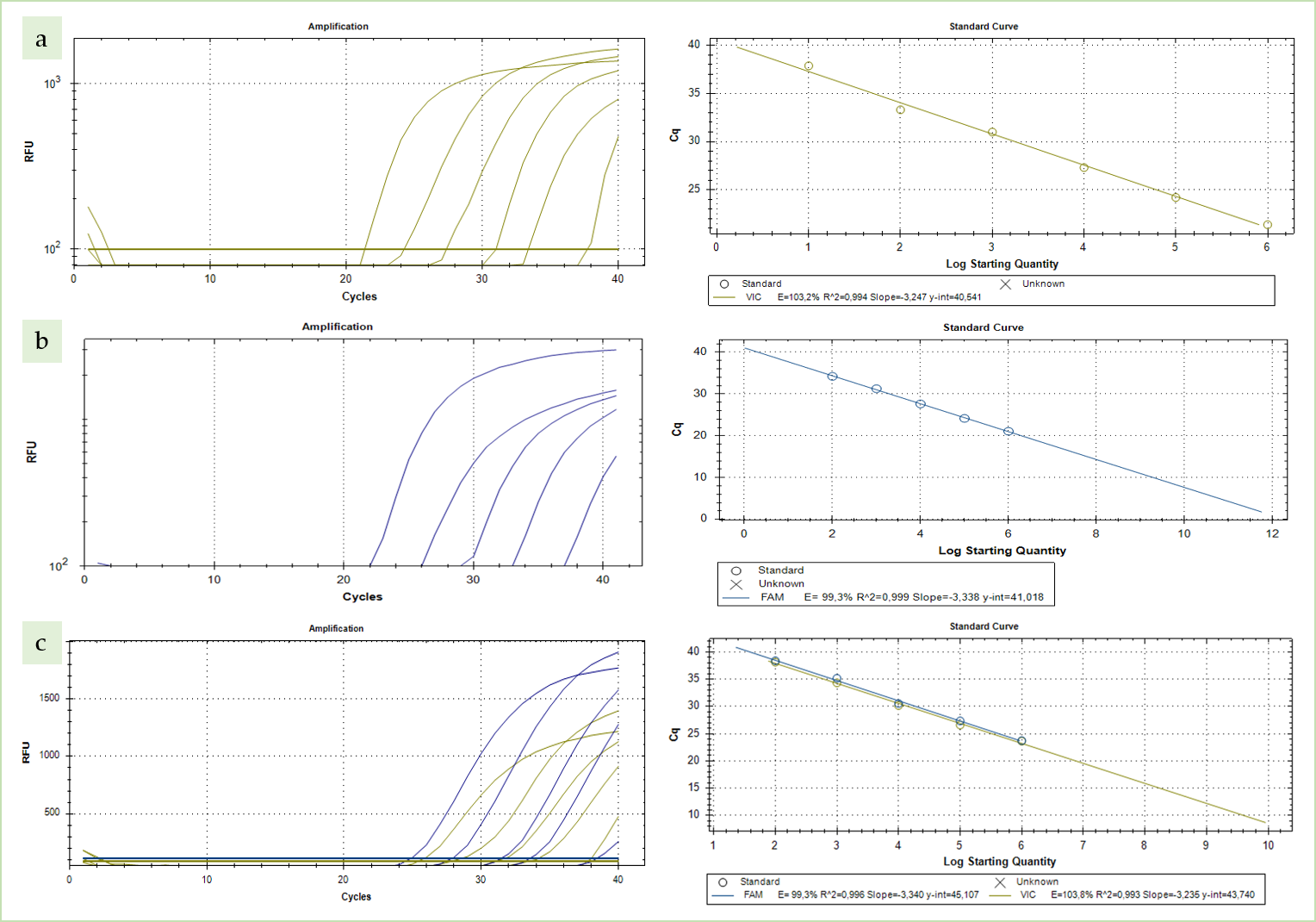

Supplement: Supplementary file 1 [file microorganisms-08-01671-s001.zip › Figure S1.tif]

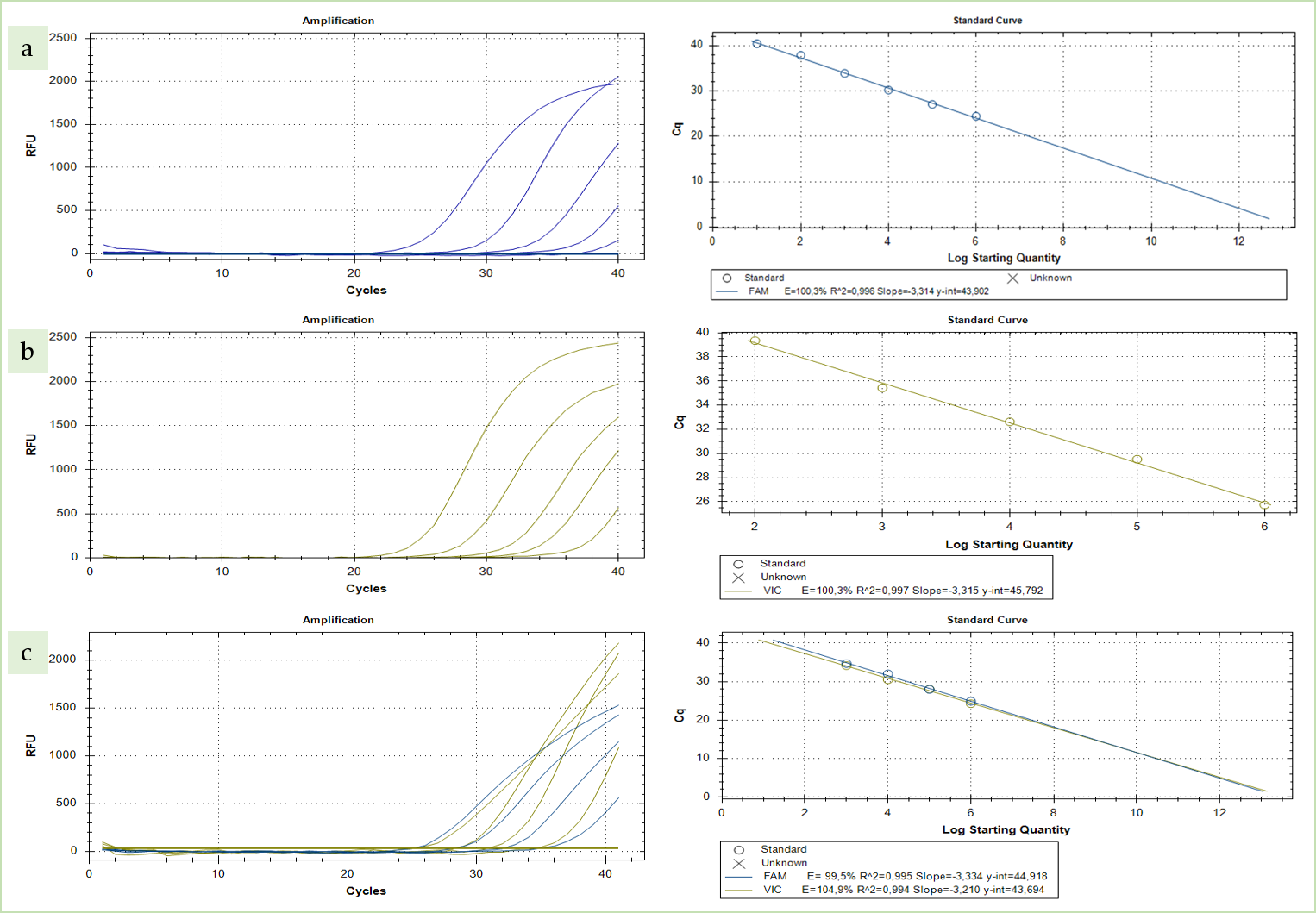

Supplement: Supplementary file 1 [file microorganisms-08-01671-s001.zip › Figure S2.tif]

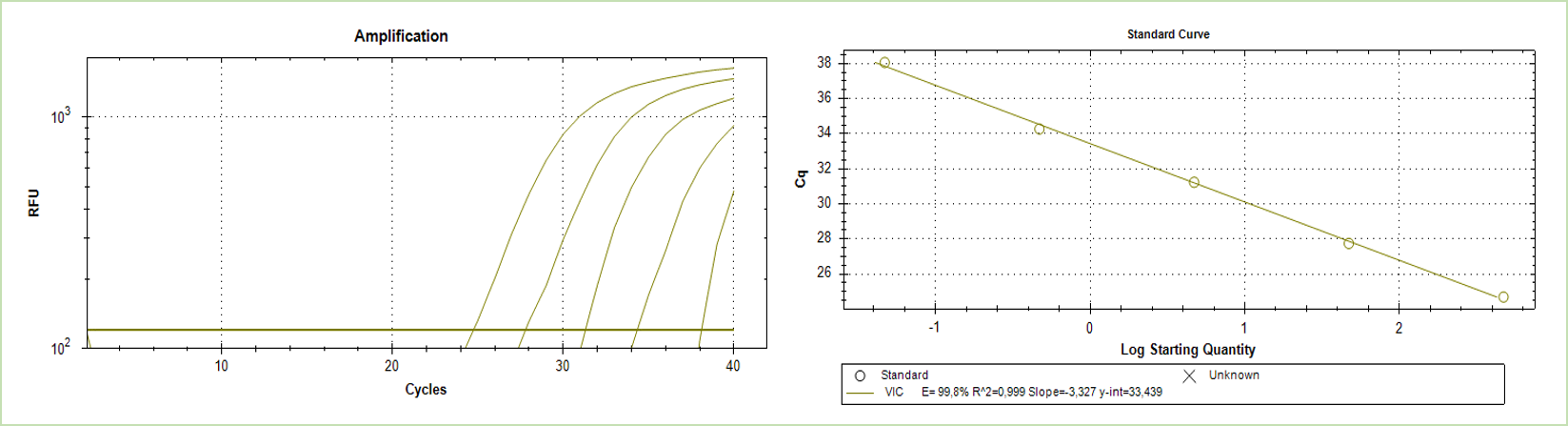

Supplement: Supplementary file 1 [file microorganisms-08-01671-s001.zip › Figure S3.tif]
